# Progressive liver failure post acute hepatitis A, over a three-month period, resulting in hepatorenal syndrome and death

**DOI:** 10.1093/gastro/gow009

**Published:** 2016-05-30

**Authors:** Tarek Turk, Tareq Al Saadi, Bisher Sawaf, Mahmoud Alkhatib, Mhd Ismael Zakaria, Bisher Daaboul

**Affiliations:** 1 Faculty of Medicine, University of Damascus, Damascus, Syrian Arab Republic; 2 Faculty of Medicine, Syrian Private University, Damascus, Syrian Arab Republic; 3 Gastroenterology and Hepatology, Medical Care Center, Damascus, Syrian Arab Republic

**Keywords:** hepatitis A virus, acute liver failure, hepatorenal syndrome

## Abstract

Hepatitis A is a common viral illness worldwide. It usually results in an acute, self-limiting disease and only rarely leads to fulminant hepatic failure or any other complications. During the period of conflict in Syria, and due to the damages to water infrastructure and poor sanitation, a dramatic increase in hepatitis A virus infection has been documented. Here we report a rare case of a 14-year-old male whose hepatitis A was complicated with hepatorenal syndrome and subacute liver failure. The war condition in Syria impeded transportation of the patient to a nearby country for liver transplantation, contributing to his unfortunate death.

## Introduction

Hepatitis A is estimated to cause about 1.4 million symptomatic cases of hepatitis annually around the globe [1]. Hepatitis A virus (HAV) infection usually results in an acute, self-limiting disease and only rarely leads to fulminant hepatic failure or any other complications [2]. Here we report a rare case of a 14-year-old male whose hepatitis A was complicated with hepatorenal syndrome and subacute liver failure resulting in his death.

### Informed consent

Informed consent was obtained from the patient's father. 

## Case Presentation

In late 2014, during an outbreak of hepatitis A in Damascus, the parents of a 14-year-old male had him tested for possible HAV infection. The parents’ worry was heightened after the diagnosis of the patient’s aunt with hepatitis A, which was complicated by acute liver failure and subsequently caused her death. The patient had abnormal liver function tests and positive hepatitis A virus antibodies. Therefore, he was diagnosed at that time with asymptomatic hepatitis. The patient was completely healthy before this diagnosis with no previous history of hepatic or immunologic diseases. Laboratory tests for hepatitis B virus and hepatitis C virus were negative. More than a month after the laboratory diagnosis was made, the patient experienced symptoms of right upper quadrant pain, fatigue, nausea, vomiting and jaundice. Lab values and antibody titers for this phase of the illness are not available.

Table 1 demonstrates the timeline of the patient’s illness through listing of the main events along with their corresponding dates. The table also contains the lab values of some important tests obtained at each corresponding event.

**Table 1.** The main events that occurred during the patient’s illness and the lab values of some important tests obtained at each corresponding event.

Time	Event	ALT (U/L)	AST (U/L)	Total bilirubin (mg/dL)	Direct bilirubin (mg/dL)	Indirect bilirubin (mg/dL)	INR	Na (mmol/L)	K (mmol/L)	Urea (mg/dL)	Creatinine (mg/dL)
0	Lab diagnosis of hepatitis A	Not available									
36 days	Symptomatic manifestations of hepatitis A	Not available									
70 days	Admission with hyponatremia	317	445	28.01	21.26	6.75	3.27	115	5.6	45	1.0
71 days	Resolution of hyponatremia and discharge	540	1026	27.5	21.5	6.0	3.40	124	4.5	56	1.0
74 days	Follow-up	350	–	28.1	16.2	11.9	–	129	3.7	37	1.1
76 days	Admission with hepatorenal syndrome	–	–	–	–	–	–	131	3.5	92	3.6
81 days	Start of terlipressin treatment	40	100	29.5	25	4.5	5.55	133	4.0	173	4.5
84 days	Development of hepatic encephalopathy and diagnosis of subacute hepatic failure	41	113	27.9	–	–	4.04	134	3.4	170	3.9
87 days	Right before death	–	–	30.75	10.26	20.49	5.03	138	4.4	182	3.58

ALT: alanine transaminase; AST: aspartate transaminase; INR: international normalized ratio.

On the 70th day after diagnosis, the patient suffered from exacerbated right upper quadrant pain, fatigue, enlarged abdomen, vomiting, drowsiness and confusion, so he was admitted to the hospital. Physical examination revealed shifting dullness, tachycardia (>130 beats/min), jaundice, muscle wasting, asterixis and bouts of agitation. Abnormal laboratory tests upon admission included elevated alanine transaminase (ALT), aspartate transaminase (AST), bilirubin and international normalized ratio (INR) (Table 1), decreased hemoglobin (10.9 g/dL) and hematocrit (32.7%). The patient also had hyponatremia and hyperkalemia (Table 1), elevated C-Reactive Protein (9.8 mg/L), hypoglycemia (50 mg/dL), and positive fecal occult blood. Analysis of a random urine sample revealed normal potassium (17.7 mmol/L) and decreased sodium (10 mmol/L) levels. Upper gastrointestinal endoscopy revealed esophageal ulcers, duodenal bulb ulcer, and portal hypertensive gastropathy. Hyponatremia and upper gastrointestinal bleeding were suspected to have caused the patient’s altered mental status. The patient was admitted to the intensive care unit (ICU) and was managed with intravenous (IV) 0.9% NaCl, IV ceftriaxone, oral laxative (lactulose), oral beta-blockers (nadolol) and hydration. In the next day, the patient’s condition improved, and he was discharged from the hospital.

On the 76th day after diagnosis, the patient developed increasing ascites and pedal and testicular edema and was admitted to the hospital for further evaluation. Abdominal multi-slice CT scan without contrast revealed excessive ascites and bilateral hydrothorax. Marked increase in levels of urea and creatinine (Table 1) with decreased urine sodium levels (9 mEq/L) was noticed at the time, and the patient was diagnosed with type 1 hepatorenal syndrome. IV albumin was added to the treatment. Five days later, the patient started taking terlipressin, a vasopressin analog, as a possible therapy for hepatorenal syndrome. A mild symptomatic improvement was noted during the next few days.

During his hospital stay, one of the patient’s routine tests revealed a high white blood cell (WBC) count (38 700, mainly lymphocytes). An extensive infectious workup including blood cultures, urinalysis and ascitic paracentesis and analysis did not yield any abnormalities. Chest x-ray revealed pleural effusion, so he underwent a diagnostic thoracic paracentesis of the previously discovered pleural effusion. Pleural fluid analysis revealed 100 WBCs per ml (mostly lymphocytes) and negative culture. IV lines were changed, and vancomycin and meropenem were added to the drugs of the patient. During the subsequent days, his WBC count went back to normal. The patient also developed watery diarrhea on the 83rd day after diagnosis of hepatitis A and was prescribed oral metronidazole.

He developed confusion, agitation, seizures and convulsions, indicating the presence of hepatic encephalopathy. He also suffered from extensive vomiting that lead to upper GI bleed due to Mallory-Weiss syndrome. The diagnosis of subacute hepatic failure was made at the time, and the patient was moved to the ICU where he was placed on an automatic ventilator due to decreased PaO_2_. The next day, he suffered from aspiration pneumonia as a result of vomiting. Two days later, 87 days after the diagnosis of hepatitis A, the patient experienced sudden cardiac arrest and expired despite multiple resuscitation attempts.

During his hospital stay, the patient received IV vitamin K and multiple units of plasma, platelets and red blood cells. During that period, the patient’s brother was also diagnosed with hepatitis A. Unlike his sibling, however, the infection resolved completely with no complications. The patient was not evaluated for autoimmune disorders or for predisposing immunologic factors, and he did not have any symptoms suggesting such diseases. Because he was completely healthy and did not have any previous history of a hepatic disease before his current illness, the presence of an underlying chronic liver disease was ruled out.

## Discussion

HAV infection usually results in a self-limiting disease that ends with recovery. Most of the symptomatic cases are among adults, whereas more than 70% of children under 6 years old are asymptomatic or experience mild symptoms [3].

HAV infection is frequent in Syria. A study in 2000 reported 89% seroprevalence of hepatitis A in the study population [4]. The study concluded that HAV infection was acquired mostly during childhood. Although HAV vaccine was recommended [5], there is currently no official HAV vaccination program in Syria. In mid 2014, the World Health Organization (WHO) warned of ‘dramatically increased risks’ of diseases that can be communicable by water including hepatitis A. This is due to damage to the water infrastructure and poor sanitation that was caused by the ongoing conflict in Syria [6]. Reports showed 31 460 cases of hepatitis A in 2014 in Syria [7], with 1056 reported cases during a 15-day period [6]. In February 2015, Dr. Elizabeth Hoff, WHO representative in Syria, stated that more than 1000 cases per week have been reported since January [8].

The patient in this case may have been infected during this outbreak in Damascus. He was in contact with his aunt, who died as a consequence of hepatitis A infection earlier. His sibling was also infected later; however, he survived the infection. The three cases of hepatitis A in this family reflect the huge incidence in the Damascus population during that period.

Acute liver failure (ALF) (i.e. fulminant hepatic failure) is defined as severe and sudden liver dysfunction leading to coagulopathy and hepatic encephalopathy in previously healthy patients [9]. Since children do not experience hepatic encephalopathy until the late stages, the diagnosis of pediatric ALF incorporates those with advanced coagulopathy regardless of mental status [10].

ALF resulting from HAV infection is rare [2]. Hepatitis A is a common cause of ALF in endemic areas [10], where it accounts for 80% of pediatric ALF [11]. However, due to low rate of infection in the developed countries, hepatitis A accounts for only 0.8% of cases of pediatric ALF [12]. Mortality reaches 90% in adults and 74% in children who do not undergo liver transplant. With liver transplantation, however, the survival rate in children with ALF may reach 80% [13]. In adults, King’s College Hospital criteria (KCHC) are commonly used to indicate the need for liver transplantation in ALF. However, it is not beneficial for children [14].

O’Grady *et al.* suggested a classification that categorizes liver failure into three subclasses: hyperacute (onset within 1 week), acute (between 8 and 28 days), and subacute (between 29 days and 12 weeks) [15]. This classification depends on the time interval between jaundice appearance and encephalopathy occurrence and indicates the survival rate for patients, giving patients with hyper-acute liver failure the best prognosis.

Our patient was admitted to the hospital with symptoms of hyponatremia and later developed hepatorenal syndrome as a complication of the hepatitis A. Three days before his death (48 days after the beginning of jaundice), he developed hepatic encephalopathy, indicating the presence of an even more serious complication: subacute liver failure.

A study in the United Kingdom revealed that a prolonged interval between the first manifestation of liver disease and the onset of encephalopathy was associated with increased likelihood of death or requirement of hepatic transplantation [16]. That period was 48 days in this case. ALF usually results in encephalopathy, which then leads to death due to brain edema and intracranial hypertension [17].

Usual manifestations of renal involvement in HAV infection are mild proteinuria, microscopic hematuria and slight urinary sediment abnormalities [18]. Hepatorenal syndrome (HRS) is idiopathic acute renal failure that occurs in patients with acute liver failure or chronic liver disease [19]. The diagnosis should be made according to the International Ascites Club criteria [20].

There are two types of HRS: type 1 is defined by doubling of the serum creatinine to reach >2.5 mg/dL during <2 weeks period, while type 2 is characterized by a slower progression to a serum creatinine of > 1.5 mg/dl [19]. Untreated HRS type 1 is associated with a bad prognosis, with mortality reaching 80% in 2 weeks and a rate of 10% surviving more than 3 months. However, untreated HRS type 2 has a better prognosis with median survival reaching 6 months [21]. The acute deterioration in our patient's condition, the increase in his creatinine levels and the decrease in his urine sodium levels increased the suspicion for type 1 HRS, and the diagnosis was made after exclusion of hypotension, proteinuria and nephrotoxic medications use. Treatment improves survival rates to reach 20% and 40% after 3 months for HRS type 1 and HRS type 2, respectively [22].

In adults with ascites and cirrhosis, incidence of HRS is approximately 8% per year [23]. It has a prevalence of 48% among those with advanced liver disease awaiting liver transplant [24]. Vasopressin analogs such as terlipressin are the first-line treatment in managing type 1 HRS [19]. Terlipressin was shown to be effective in the treatment of HRS type 1, with potential survival benefits [25]. Liver transplant is the choice for those who do not respond to vasopressor therapy and who require renal support [19].

Our patient showed a mild symptomatic improvement from the treatment with albumin and terlipressin. He died before a liver transplant could be performed. The delay in liver transplantation was mainly due to lack of specialized centers for it in Syria and the difficulties that faced the parents in transferring their son to a nearby country due to the war condition.

## Conclusion

The complications of hepatorenal syndrome and acute liver failure are very rare in the context of hepatitis A infection. However, during the complications of the Syrian war, it appears to be much more challenging to manage such patients.

*Conflict of interest statement:* none declared.
